# The Multifaceted MEP Pathway: Towards New Therapeutic Perspectives

**DOI:** 10.3390/molecules28031403

**Published:** 2023-02-01

**Authors:** Alizée Allamand, Teresa Piechowiak, Didier Lièvremont, Michel Rohmer, Catherine Grosdemange-Billiard

**Affiliations:** 1 Laboratoire de Chimie et Biochimie de Molécules Bioactives—Université de Strasbourg/CNRS, UMR 7177, Institut Le Bel, 4 Rue Blaise Pascal, 67081 Strasbourg, France; 2 Unité de Chimie des Biomolécules, Institut Pasteur, Université Paris Cité, CNRS UMR3523, 75724 Paris, France

**Keywords:** antibacterial drug, immunostimulant, isoprenoids, laurencione, MEP pathway, phosphoantigen, prodrug, quorum sensing

## Abstract

Isoprenoids, a diverse class of natural products, are present in all living organisms. Their two universal building blocks are synthesized via two independent pathways: the mevalonate pathway and the 2-*C*-methyl-ᴅ-erythritol 4-phosphate (MEP) pathway. The presence of the latter in pathogenic bacteria and its absence in humans make all its enzymes suitable targets for the development of novel antibacterial drugs. (*E*)-4-Hydroxy-3-methyl-but-2-enyl diphosphate (HMBPP), the last intermediate of this pathway, is a natural ligand for the human Vγ9Vδ2 T cells and the most potent natural phosphoantigen known to date. Moreover, 5-hydroxypentane-2,3-dione, a metabolite produced by *Escherichia coli* 1-deoxy-ᴅ-xylulose 5-phosphate synthase (DXS), the first enzyme of the MEP pathway, structurally resembles (*S*)-4,5-dihydroxy-2,3-pentanedione, a signal molecule implied in bacterial cell communication. In this review, we shed light on the diversity of potential uses of the MEP pathway in antibacterial therapies, starting with an overview of the antibacterials developed for each of its enzymes. Then, we provide insight into HMBPP, its synthetic analogs, and their prodrugs. Finally, we discuss the potential contribution of the MEP pathway to quorum sensing mechanisms. The MEP pathway, providing simultaneously antibacterial drug targets and potent immunostimulants, coupled with its potential role in bacterial cell–cell communication, opens new therapeutic perspectives.

## 1. Introduction

Isoprenoids represent a diverse family of natural products. They are present in all living organisms as essential primary metabolites (e.g., sterols modulating fluidity and permeability of eukaryotic membranes, carotenoids in photosystems of phototrophic organisms, prenyl chains of quinones involved in electron transport systems, or polyprenol derivatives serving as carbohydrate transporters) and as specialized metabolites of less obvious biological significance (e.g., mono- and diterpenes from essential oils, triterpenes, and meroterpenoids where a terpenoid is linked to a non-terpenic moiety).

**Scheme 1:**
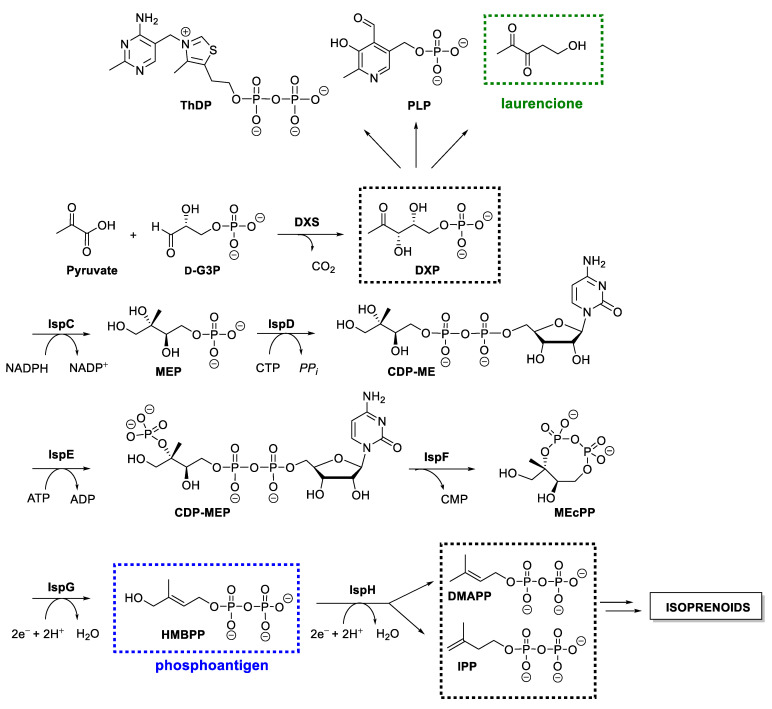
The multifaceted MEP pathway. ThDP: thiamine diphosphate; PLP: pyridoxal phosphate; ᴅ-G3P: ᴅ-glyceraldehyde 3-phosphate; DXS: 1-deoxy-ᴅ-xylulose 5-phosphate synthase; DXP ; IspC: 1-deoxy-ᴅ-xylulose 5-phosphate reductoisomerase; MEP: 2-*C*-methyl-ᴅ-erythritol 4-phosphate; IspD: 2-*C*-methyl-ᴅ-erythritol 4-phosphate cytidyl transferase; CDP-ME: 4-diphosphocytidyl-2-*C*-methyl-ᴅ-erythritol; IspF: 2-*C*-methyl-ᴅ-erythritol 2,4-cyclodiphosphate synthase; MEcPP: 2-*C*-methyl-ᴅ-erythritol-2,4 cyclodiphosphate; IspG: 2-*C*-methyl-ᴅ-erythritol 2,4-cyclodiphosphate reductase; HMBPP: (*E*)-4-hydroxy-3-methyl-but-2-enyl diphosphate; IspH: 4-hydroxy-3-methylbut-2-enyl diphosphate reductase; DMAPP: dimethylallyl diphosphate; IPP: isopentenyl diphosphate.

Despite their diversity, isoprenoids are synthesized in all living organisms through the assembling of the same two universal C_5_ building blocks, namely isopentenyl diphosphate (IPP) and dimethylallyl diphosphate (DMAPP) (Scheme 1). Their biosynthesis occurs in different taxons via two distinct and independent pathways. The mevalonate pathway discovered in the 1950s and starting from acetyl coenzyme A is the sole source of the isoprene units in animals, fungi, and the cytoplasm of all phototrophic eukaryotes, in a few bacteria, and in Archaea. In contrast, an alternative mevalonate-independent route, the 2-*C*-methyl-ᴅ-erythritol pathway (Scheme 1), discovered in the mid-1990s, is present in all phototrophic organisms (plants and algae) and phylogenetically related taxa (e.g., *Plasmodium* spp.), though its presence in plants is restricted to the plastids, as well as in nearly all bacteria, including many pathogens [1,2,3]. Starting from pyruvate and ᴅ-glyceraldehyde 3-phosphate (ᴅ-G3P), the first step of the pathway catalyzed by the 1-deoxy-ᴅ-xylulose 5-phosphate synthase (DXS) gives 1-deoxy-ᴅ-xylulose 5-phosphate (DXP) [4], a branching point metabolite also required for the biosynthesis of essential cofactors such as pyridoxal phosphate (PLP) (vitamin B6) [5,6] and thiamine diphosphate (ThDP) (vitamin B1) [7,8]. The conversion of DXP into 2-*C*-methyl-ᴅ-erythritol 4-phosphate (MEP), the key intermediate of this pathway, is catalyzed by the 1-deoxy-ᴅ-xylulose 5-phosphate reductoisomerase (DXR/IspC). The next steps consist of MEP modifications and activation catalyzed by the 2-*C*-methyl-ᴅ-erythritol 4-phosphate cytidyl transferase (IspD), the 4-(cytidine 5′-diphospho)-2-*C*-methyl-ᴅ-erythritol kinase (IspE) and the 2-*C*-methyl-ᴅ-erythritol 2,4-cyclodiphosphate synthase (IspF), followed by two reduction steps catalyzed by the 2-*C*-methyl-ᴅ-erythritol 2,4-cyclodiphosphate reductase (IspG) and the 4-hydroxy-3-methylbut-2-enyl diphosphate reductase (IspH), respectively [4].

The absence of the MEP pathway in humans and its presence in many human pathogens such as *Pseudomonas*, *Klebsiella*, and *Mycoplasma,* as well as *Toxoplasma* and *Plasmodium* species, make all its enzymes attractive targets for designing original antimicrobials and therefore have spurred intense research worldwide [9].

In 2001, the last intermediate of the MEP pathway, the (*E*)-4-hydroxy-3-methyl-but-2-enyl diphosphate (HMBPP), was identified to activate Vγ9Vδ2 T cells, which play a crucial role in the immune system [10]. Thus, it is interesting to note that the MEP pathway is essential for the viability of human pathogens and concurrently triggers an immune response inducing their death.

Moreover, the 5-hydroxypentane-2,3-dione (laurencione, Scheme 1), a bacterial metabolite produced by *Escherichia coli* DXS [11], the first enzyme of the MEP pathway, from free ᴅ-glyceraldehyde and from pyruvate, exhibits a close structural resemblance to a small signal molecule, (*S*)-4,5-dihydroxy-2,3-pentanedione (DPD). This suggests an unsuspected role of the MEP pathway in the mechanisms of bacterial cell communication [12].

The present work gives a brief overview of the multifaceted roles of the MEP pathway as (i) a highly selective antibacterial target, (ii) the most potent known phosphoantigen source, and (iii) a potential contributor to quorum sensing (QS) mechanisms.

Finally, we discuss the new perspectives opened by the MEP pathway functional duality as well as new cryptic functions related to the QS.

## 2. Antibacterials

Today, antimicrobial resistance (AR) and healthcare-associated infections (HCAIs) can be referred to as a silent pandemic and are among the world’s most pressing public health problems, leading to increase patient morbidity and mortality, thus becoming a significant economic concern. Numerous microorganisms associated with these HCAIs and ARs include mainly Gram-negative bacteria (e.g., *Neisseria gonorrhoeae*, *Escherichia coli*, *Klebsiella pneumoniae*, *Pseudomonas aeruginosa*, *Enterococcus faecium*, and *Acinetobacter* spp.) but also some Gram-positive bacteria (e.g., *Staphylococcus aureus*). To treat bacterial infections, the majority of currently used antibiotics target the biosynthesis of proteins, nucleic acids, and cell wall constituents. In addition, the antibiotics introduced to the market in recent decades are analogs of molecules discovered in the eighties [13]. Consequently, bacteria have developed multiple resistance mechanisms leading to a drastic loss of efficiency of the current antibacterial treatments.

Therefore, there is an urgent need to find innovative therapeutic targets essential for bacterial survival for the development of new antibacterial strategies. Absent in humans, the MEP pathway is a very attractive target owing to the druggability of all its enzymes, a potential for a new class of antibiotics with expected minor side effects for humans.

### 2.1. Inhibitors of DXS

DXS is a dimeric thiamine diphosphate (ThDP)-dependent enzyme [14]. It catalyzes the first and rate-limiting step of the MEP pathway, which consists of a decarboxylative condensation of two products of glycolysis: ᴅ-G3P and pyruvate into 1-deoxy-ᴅ-xylulose 5-phosphate (DXP). The diverse roles of DXP as a precursor not only to isoprenoid precursors IPP and DMAPP but also to ThDP and PLP make DXS an attractive antibacterial target [15,16]. Nonetheless, only a small number of DXS inhibitors have been reported so far, probably due to the lack of available structural information on this enzyme.

**Figure 1:**
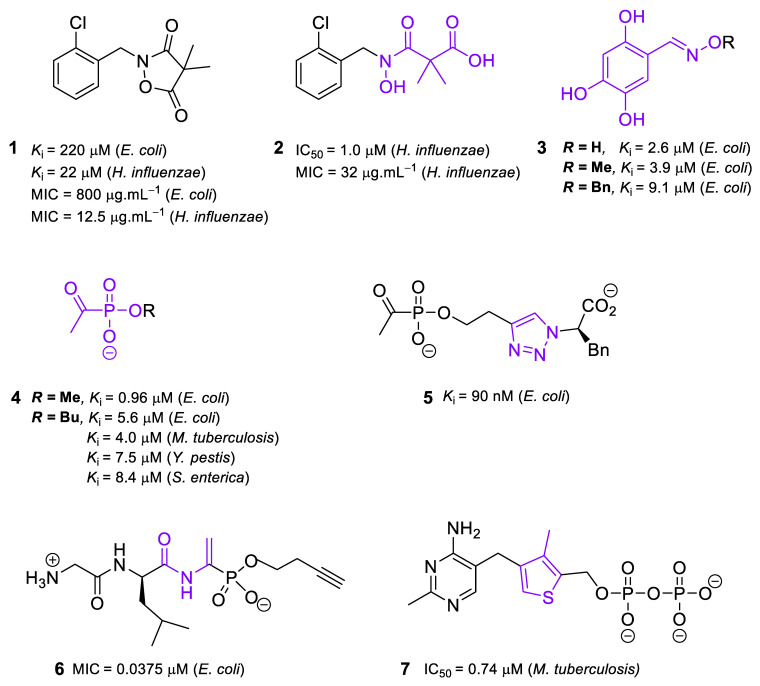
Representative inhibitors of DXS. IC_50_: half maximal inhibitory concentration; MIC: minimum inhibitory concentration; *K*_i_: inhibitory constant.

The first reported DXS inhibitors were a commercial herbicide ketoclomazone **1** and its hydrolyzed derivative **2**. (Figure 1) [17,18]. Both inhibit the DXS of *Haemophilus influenzae* and *E. coli* at mid to low micromolar concentration in an uncompetitive manner with respect to pyruvate and exhibit a moderate effect on the bacterial growth of these pathogens.

A series of aromatic oxime DXS inhibitors was developed, among which 2,4,5-trihydroxybenzaldoximes **3** (Figure 1) have been identified to exhibit *K*_i_ values within the one-digit micromolar range [19]. These inhibitors are competitive against ᴅ-G3P and uncompetitive against pyruvate. However, the development of antibacterial drugs based on these compounds may be problematic as the trihydroxyphenyl moieties are readily oxidized in vivo, forming highly electrophilic and thus toxic quinones. Meyers et al. prepared a series of alkylacylphosphonates **4** (Figure 1), unnatural bisubstrate analogs including a pyruvate mimic and a nonpolar acceptor allowing the formation of irreversible complexes with ThDP in the active site of the enzyme [20,21]. These bisubstrates are selective DXS inhibitors of several pathogens (*M. tuberculosis*, *Salmonella enterica*, and *Yersinia pestis*) in the low micromolar range. Recently, a library of triazole-based alkylacylphosphonates has been reported, among which compound **5** (Figure 1) emerged as the most potent inhibitor against *E. coli* DXS (*K*_i_ = 90 nM) with a great selectivity (more than 15,000-fold) over porcine pyruvate dehydrogenase. However, due to the poor permeability of the bacterial plasma membrane towards these potential drugs, they exhibit weak antibacterial activity against Gram-negative (e.g., *E. coli*, *S. enterica*) and Gram-positive bacteria (e.g., *Bacillus anthracis)* [22]. To improve their uptake into the cells, peptidic enamide prodrugs of alkylacylphosphonates have been recently prepared, allowing an increase in their antibacterial activity against Gram-negative pathogens and decreasing their minimum inhibitory concentrations by nearly 2000-fold in comparison with free inhibitors (e.g., **6**, Figure 1) [23]. Finally, a series of 3-diazathiamine diphosphate analogs (e.g., **7**, Figure 1) widely studied as inhibitors of a large collection of ThDP-dependent enzymes has been reported to inhibit *M. tuberculosis* DXS with a sub-micromolar activity [24].

### 2.2. Inhibitors of DXR/IspC

DXR, the second enzyme of the MEP pathway, catalyzes a two-step reaction: the Mg^2+^-triggered rearrangement of DXP into a non-isolable aldehyde and its concomitant NADPH-dependent reduction into MEP. DXR is a particularly well-described enzyme of the MEP pathway, with numerous protein crystal structures from several organisms, including important pathogens (e.g., *E. coli*, *M. tuberculosis*, *Y. pestis*). Additionally, its active site was assessed as a particularly druggable pocket [9]. However, the design of DXR inhibitors is challenging due to strong conformational changes of the enzyme upon ligand binding [9] and a great dependence of the inhibitor efficiency on the bacterial species.

**Figure 2:**
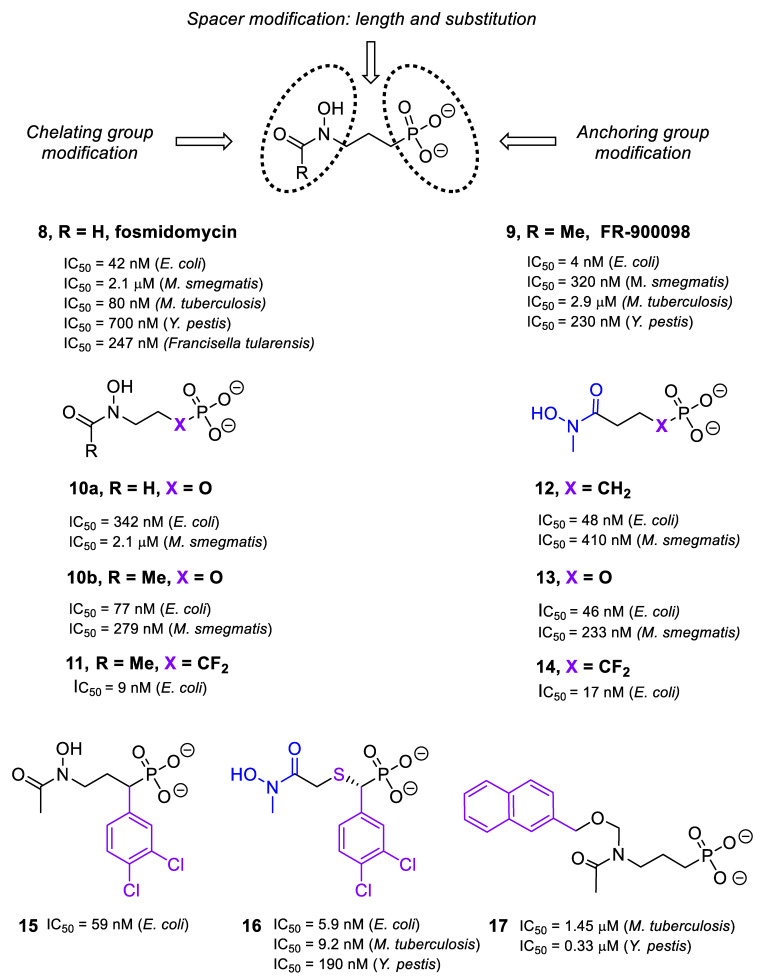
Representative inhibitors of DXR.

In 1980, fosmidomycin **8** and its acetylated analog FR-900098 **9** (Figure 2), two phosphonoretrohydroxamic acids originally isolated from *Streptomyces* spp. culture broths were shown to exhibit antibacterial activity against a wide variety of bacteria, mainly Gram-negative ones. Twenty years later, the discovery of the MEP pathway has highlighted data on the mechanism and the target of those phosphonic acid antibiotics. Fosmidomycin was shown to inhibit the DXR of *E. coli* in a mixed (competitive with respect to DXP and uncompetitive relative to NADPH), low tight-binding manner, with a *K*_i_ value of 38 nM [25,26,27].

This natural antibiotic binds to the DXR active site via hydrogen bonds between the phosphonate moiety and polar amino acid residue and chelates the divalent cation through its retrohydroxamate metal-binding group.

Although fosmidomycin **8** and its analog **9** efficiently inhibit the DXR enzyme of a wide range of bacteria, they have several limitations to being used as suitable antibacterial agents: (i) fosmidomycin-resistant strains have already appeared [28]; (ii) fosmidomycin is ineffective against some pathogens [29]; (iii) fosmidomycin exhibits fast clearance by the kidneys and a low biodisponibility to bacterial cells due to its highly polar hydrophilic nature [30]. The presence of the negative charge on the phosphonate group prevents fosmidomycin from entering the bacterial cell by passive diffusion, and consequently, the drug has to be actively transported across the membranes to reach its target, e.g., through a transporter in the *E. coli* cytoplasmic membrane (G3P transporter GlpT) or through specific porins in the *P. aeruginosa* outer membrane [31]. Fosmidomycin is therefore inefficient against pathogens such as *M. tuberculosis* lacking such an active transport.

Fosfoxacin **10a,** a phosphate analog of fosmidomycin isolated from the filtrate of a *Pseudomonas fluorescens* culture, [32] and its acetylated analog **10b** (Figure 2) inhibit DXR of a cyanobacterium *Synechocystis* sp. with *K*_i_ values of 19 and 2 nM, respectively, vs. 58 nM for fosmidomycin [33]. Fosfoxacin also inhibits the growth of different bacterial strains; in particular, good activity was observed against *Micrococcus luteus* with a minimum inhibitory concentration (MIC) value of 0.2 μg mL^−1^ [32]. The in vitro activity of fosfoxacin on the DXR of *E. coli* was, however, rather modest, with an IC_50_ value about 8 times higher than that of fosmidomycin and 85 times higher than that of FR-9000098. Nevertheless, its acetylated analog **10b** inhibits *E. coli* and *Mycobacterium smegmatis* enzymes with an efficiency similar to that of fosmidomycin [34].

Due to the emergence of numerous clinical multidrug-resistant strains, research efforts were dedicated to the development of more effective DXR inhibitors. To improve the activity of the natural phosphonic acids, several groups performed various structure modifications that can be classified into three main categories (Figure 2): (i) replacement of the retro-hydroxamate chelating group with a different metal-binding group; (ii) replacement of the phosphonate anchoring group by phosphonate bioisosters; (iii) modification of the carbon spacer (length change, introduction of an aromatic substituent, introduction of fluorine atoms, replacement of a carbon atom with a heteroatom).

These detailed structure-activity relationship studies showed that neither the retrohydroxamate nor hydroxamate chelating moiety nor the phosphate/phosphonate anchoring group can be replaced, and the length of the spacer (3 atoms) cannot be changed without a drastic loss of DXR inhibitory activity [33,35,36,37].

The synthetic analog of fosmidomycin **12**, in which the retrohydroxamate moiety is inversed into hydroxamate (Figure 2) [26], inhibited DXR of *E. coli* nearly as efficiently as fosmidomycin with a *K*_i_ value of 54 nM and inhibited *M. smegmatis* DXR almost as efficiently as FR-900098. In addition, it suppressed the growth of this bacterium at 50 µM and the growth of an *E. coli* fosmidomycin-resistant strain at 200 µM [26,38]. Its phosphate analog **13** (Figure 2) had a very similar IC_50_ value for the *E. coli* enzyme and an even lower value for the *M. smegmatis* DXR. It also presented an antibacterial activity similar to those of fosfoxacine **10a** and its acetylated homolog **10b** against *E. coli* [39].

To mimic the phosphate group and increase the bioavailability, the substitution of the α,α-methylene group in phosphonate DXR inhibitors with electron-withdrawing substituents, in particular fluorine atoms, has been considered. The α,α-difluoromethylene, an isopolar mimic of the oxygen component of the P–O–C linkage in phosphates, favors the DXR inhibition and enhances the antimicrobial activity compared to the phosphates and non-fluorinated phosphonates. For example, the *N*-methylated α,α-difluorophosphonates **11** and **14** (Figure 2) exhibited stronger inhibition activity than that of the reference compound fosmidomycin against *E. coli* DXR with IC_50_ values of 9 nM and 17 nM, respectively. Moreover, a direct relationship between the capacity to inhibit the DXR and the bacterial growth was observed for α,α-difluorinated inhibitors [40].

The influence of aromatic substituents in the α-position respective to the fosmidomycin phosphonate group has also been investigated. A series of analogs was prepared, and their activity was evaluated on *E. coli* DXR [9,41]. The 3,4-dichlorophenyl fosmidomycin analog **15** (Figure 2) emerged as the most potent analog of this series with a submicromolar inhibitory activity against *E. coli* DXR (IC_50_ = 59 nM) [42]. The substitution of the methylene group in the β-position relative to the phosphonate group of **12** with a sulfur atom resulted in a dramatic improvement of the inhibitory efficiency. Indeed, compound **16** (Figure 2) exhibited IC_50_ values almost two orders of magnitude lower than fosmidomycin with respect to the enzymes of *E. coli* and *M. tuberculosis* [43]. This compound also inhibited the DXR of *Y. pestis* with a potency about three times higher than that of fosmidomycin, and it suppressed the growth of this pathogen with a similar efficiency [44].

Unlike conventional DXR inhibitors, compound **17** (Figure 2), containing an *O*-linked naphthyl moiety, which cannot efficiently chelate the metal cation in the active site of the enzyme, displayed a more potent inhibition against *Y. pestis* DXR (IC_50_ = 0.35 µM) than against its *M. tuberculosis* homolog (IC_50_ = 1.45 µM). This type of compound may occupy both DXP and NADPH binding sites and acts as a competitive bisubstrate inhibitor of DXR with respect to both DXP and NADPH. However, **17** does not appreciably suppress the *E. coli* and *Y. pestis* growth, partially because of an active efflux [45].

An important challenge in antibacterial drug development is the discovery of new antitubercular medicines, as the current treatment of tuberculosis is lengthy, associated with unpleasant side effects, and often ineffective due to the emergence of resistant *M. tuberculosis* strains [27]. To solve this problem, targeting the enzymes of the MEP pathway, especially DXR, is often attempted.

However, fosmidomycin, FR-900098, and many of their analogs [27] do not suppress the growth of *Mycobacterium* spp. despite their activity towards mycobacterial DXR. This is caused by the lack of uptake as *Mycobacterium* spp. are characterized by an extremely thick and hydrophobic cell wall rich in mycolic acids [37] and *glpT* homolog gene in the *M. tuberculosis* genome [46,47].

**Figure 3:**
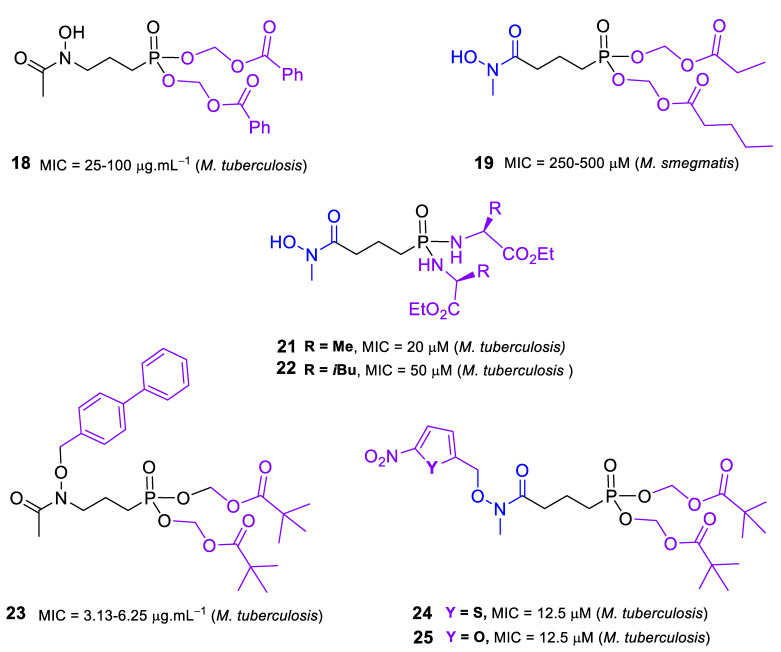
Representative prodrug of fosmidomycin analogs.

To overcome the absence of mycobacterial cell wall crossing by the natural DXR inhibitors **8** and **9** as well as by the synthetic *N*-methylated phosphonohydroxamate **12**, the prodrug strategy was adopted. Several prodrugs of fosmidomycin and its analogs were synthesized by masking the negative charge of the phosphonate group present at physiological pH with hydrophobic groups such as acyloxymethyl and alkoxycarbonyloxymethyl moieties. Among such lipophilic esters of FR-900098, compound **18** (Figure 3) has been shown to be the most potent anti-*M. tuberculosis* activity, inhibiting the growth of *M. tuberculosis* with a MIC between 25 and 100 μg mL^−1^ [48]. A similar result was observed with its acyloxymethyl phosphonate ester prodrug analog **19** (Figure 3), with a MIC value against *M. smegmatis* between 250 and 500 μM. The parent molecules are liberated inside the bacterial cells by non-specific esterases [38].

Recently, another successfully tested approach on *M. tuberculosis* was the use of phosphonodiamidate prodrugs derived from amino acid esters. Among the series, the ethyl l-alanine and ethyl l-leucine diamidates **21** and **22**, respectively (Figure 3), displayed moderate antitubercular activity with MIC of 20 and 50 μM, respectively. The liberation of the parent compound is putatively driven by two enzymatic steps catalyzed by a carboxypeptidase and a phosphoramidase [49].

To increase the lipophilicity of *O*-linked aromatic inhibitors such as **17** (Figure 2), their bis-pivaloyloxymethyl (POM) ester prodrugs were prepared and have shown improved efficiency against *M. tuberculosis*, but no activity against *E. coli* and *Y. pestis* was observed due to their active efflux. Compound **23** (Figure 3) efficiently inhibited the growth of *M. tuberculosis* with a MIC value varying according to the media used: 12.5 µg mL^−1^ (7H9 media) or between 3.13 and 6.25 µg mL^−1^ (GAST-Fe media) [45].

In search for new antitubercular compounds, a series of eleven double prodrugs of the phosphonohydroxamate **12** (Figure 2), in which the phosphonate group is masked with a POM moiety and the hydroxamate with various functional groups (ether, ester, carbamate, and bioreducible nitro aromatic group), were prepared. Only double prodrugs **24** and **25** (Figure 3) displayed promising antitubercular activity with a MIC value of 12.5 µM [50].

### 2.3. Inhibitors of IspD/YgbP

The third enzyme of the MEP pathway is a cytidyl transferase catalyzing the transfer of the cytidyl phosphate group from cytidine triphosphate (CTP) to the phosphorus atom of MEP, leading to 4-diphosphocytidyl-2-*C*-methyl-ᴅ-erythritol (CDP-ME) and inorganic diphosphate [51,52].

The design of substrate-competitive IspD inhibitors is challenging, as its active site is rather solvent-exposed and the least lipophilic of all the enzymes of the MEP pathway [9,53]. Moreover, the presence of a homologous cytidyl transferase in human cells, hISPD, may prevent the application of discovered inhibitors in therapy [54]. Most of the literature on the inhibition of IspD concerns herbicides and antimalarials [9]

**Figure 4:**
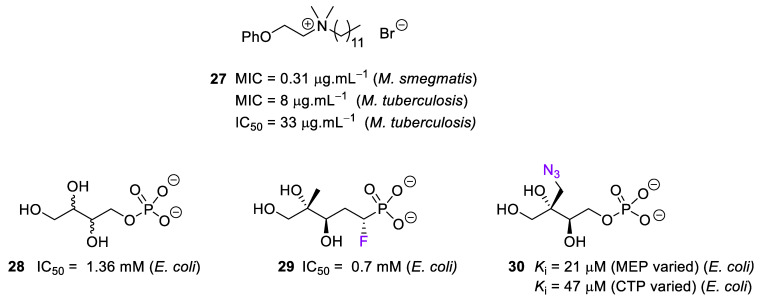
Representative inhibitors of IspD.

Domiphen bromide **27** (Figure 4), an antiseptic quaternary ammonium salt [55], inhibited the IspD of *M. tuberculosis* with an IC_50_ value of 33 μg mL^−1^ and suppressed the growth of *M. smegmatis* and *M. tuberculosis* with a MIC of 0.31 μg mL^−1^ and 8 μg mL^−1^ respectively, without appreciable loss of activity against clinical drug-resistant isolates of the latter species. It is a competitive IspD inhibitor with respect to CTP and uncompetitive with respect to MEP [56].

Antibacterials targeting the MEP pathway could also function as alternative substrates of its enzymes. Such small molecules would be transformed into nonfunctional analogs of isoprenoid precursors, hence suppressing the isoprenoid production in the bacterial cell. Inhibitors **28** and **29** are such compounds [57].

The demethylated analog of MEP **28** (Figure 4) is a weak competitive inhibitor of *E. coli* IspD with an IC_50_ value of 1.36 mM, and it reduced the turnover rate of this enzyme compared to the natural substrate [58,59]. The fluoro analog of MEP **29** is a very weak inhibitor of the *E. coli* IspD with an IC_50_ value of 0.7 mM, but no activity was found against the enzyme of *M. tuberculosis*. In addition, *E. coli* IspD catalytic efficiency is 250 times lower with **29** than that of the natural substrate [57].

MEPN_3_ **30**, an azido analog of MEP (Figure 4), is a mixed-type inhibitor of IspD with respect to both of its substrates, and it may occupy different pockets in the active site. It is characterized by two-digit micromolar *K*_i_ values against the enzyme of *E. coli* [60].

It is interesting to note that fosmidomycin **8** (Figure 2), the potent inhibitor of DXR, also weakly inhibits IspD, with an IC_50_ value of 20.4 mM for the enzyme of *E. coli*, and that the overexpression of IspD confers fosmidomycin resistance in this bacterium [61].

### 2.4. Inhibitors of IspE

The product of IspD, 4-diphosphocytidyl-2-methylerythritol, undergoes the ATP-dependent phosphorylation to 4-diphosphocytidyl-MEP (CDP-MEP). This reaction is catalyzed by IspE, a cytoplasmic kinase originally called YchB. IspE is an Mg^2+^-dependent [62] kinase belonging to the galactose, homoserine, mevalonate, and phosphomevalonate (GHMP) kinases superfamily, which also encompasses enzymes of the mevalonate pathway [4].

Although kinases related to IspE are present in mammalian cells, it was suggested that distinctive features of the IspE active site should allow its selective targeting, even if its low lipophilicity constitutes an additional challenge for the design of its inhibitors [9]. A high degree of conservation of the amino acids in the active site should enable the design of broad-spectrum antibacterials [9,63]. However, drastic differences in activity with respect to IspE from different bacterial species were observed for some inhibitors [64].

**Figure 5:**
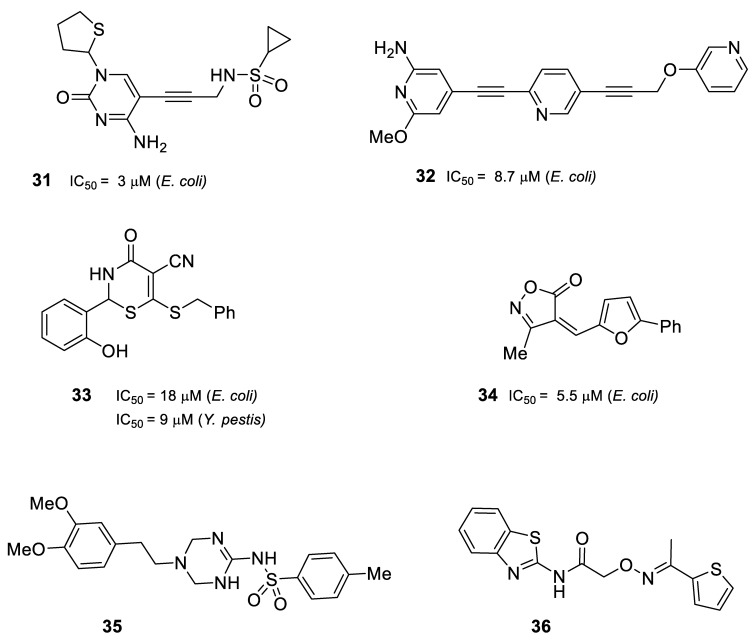
Representative inhibitors of IspE.

With the aim to obtain selective inhibitors of IspE, the possibility to simultaneously occupy the cytosine-binding pocket in the active site and the neighboring hydrophobic pocket was investigated with substituted cytosine mimics. Competitive and mixed micromolar and submicromolar inhibitors of *E. coli* IspE were obtained. Among this series, the racemic mixture of **31** (Figure 5) was characterized by the lowest *K*_i_ value (290 nM) [65].

Compound **32** (Figure 5), presumably bisubstrate inhibitor of IspE targeting both the 4-diphosphocytidyl-2-methylerythritol- and the ATP-binding sites, has a similar efficiency as **31** [66].

Investigation of known inhibitors of the GHMP kinases superfamily led to the discovery of two new hits for the development of the IspE inhibitors, compounds **33** and **34** (Figure 5). They were characterized by one- or low two-digit micromolar activities against the enzymes of *E. coli* and *Y. pestis* [9,67]. Moreover, **33** suppressed the *E. coli* growth with an activity similar to that of antiseptic hexachlorophene during the first 6 h of the culture, although its effect was drastically reduced later on [67]. In the same study, ten weak *E. coli* IspE inhibitors were identified by high-throughput in silico screening. The most active of them was compound **35** (Figure 5), which inhibited the enzyme activity by 80% at 20 μM. However, compound **36** (Figure 5), with only a 60% inhibition at the same concentration range, was judged more interesting for further optimization because of the originality of its structure [67].

### 2.5. Inhibitors of IspF

The formation of 2-*C*-methyl-ᴅ-erythritol-2,4 cyclodiphosphate (MEcPP) is catalyzed by IspF, the fifth enzyme of the MEP pathway. The activity of this trimeric enzyme depends on two metal cations: Zn^2+^ and Mg^2+^ or Mn^2+^ [4]. The Zn^2+^ ion coordinates the phosphate group bound to the C4 atom of the substrate [68], increasing its electrophilicity and facilitating its nucleophilic attack at the C2 atom. The resulting intermediate undergoes cyclization, yielding MEcPP and cytidine monophosphate (CMP) [4].

IspF might play an important role in the regulation of the MEP pathway as it may be inhibited by downstream isoprenoid precursors. Structural analysis elucidated the presence of a hydrophobic cavity at the core of the trimer where IPP, DMAPP, geranyl diphosphate (GPP), and farnesyl diphosphate (FPP) were found to bind. However, none of them inhibits IspF. Nevertheless, Bitok and Meyers observed that MEP and 2-C-methyl-ᴅ-erythritol (ME) enhanced IspF activity and stability. Afterward, only FPP turned out to inhibit this IspF-MEP complex, probably binding the hydrophobic cavity. This suggests that MEP binding induces a conformational change in IspF. Thus, feedback regulation of the MEP pathway may occur via the IspF-MEP complex inhibition [69,70].

In addition, the FPP- and GPP-binding hydrophobic cavity of the IspF trimer was assessed as druggable. However, few IspF inhibitors have been described [9]. Therefore, it is interesting to note that interactions of this enzyme with inhibitors may depend on the binding of its allosteric regulators, such as MEP [71].

**Figure 6:**
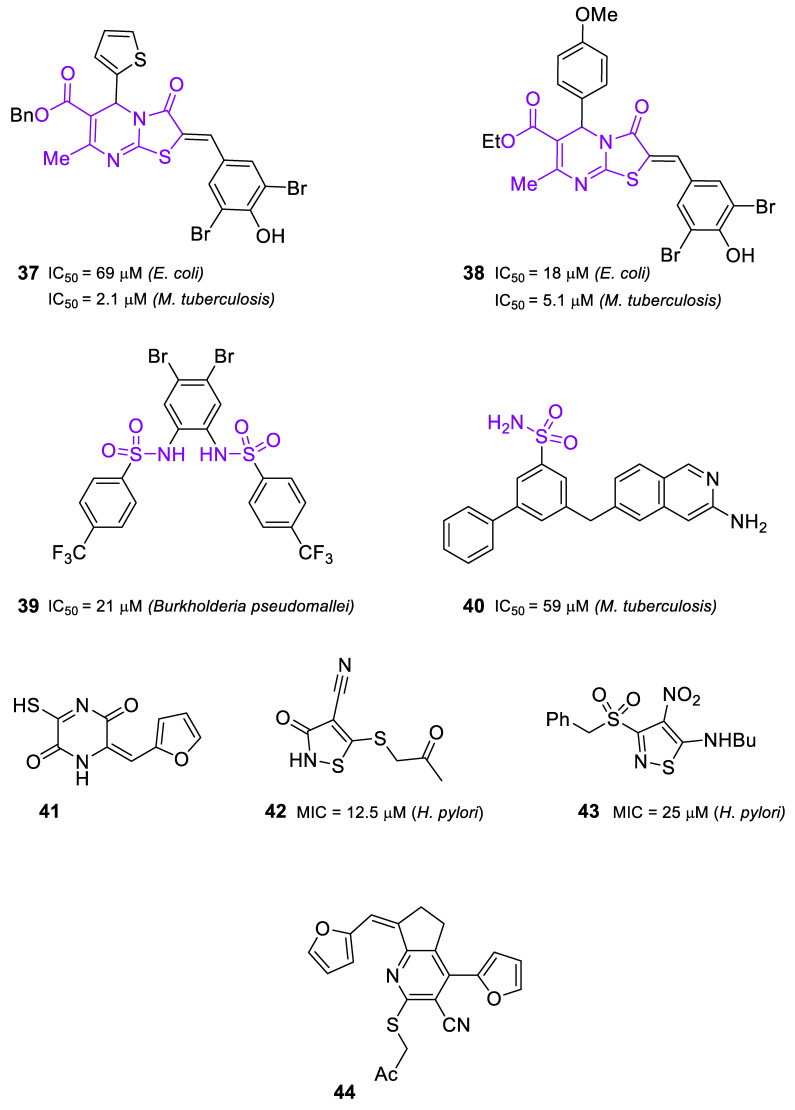
Representative inhibitors of IspF and IspDF.

High-throughput screening led to the identification of thiazolopyrimidines as IspF inhibitors. Among the most potent molecules identified, **37** and **38** (Figure 6) were characterized by IC_50_ values in the low micromolar range: 2.1 µM and 5.1 µM, respectively, against the IspF of *M. tuberculosis* and 69 µM and 18 µM, respectively, against the IspF of *E. coli* [72].

A few two-digit micromolar inhibitors of *Burkholderia pseudomallei* IspF were identified, among which bis-sulfonamide **39** (Figure 6) is the most potent. The aryl sulfonamide **40**, designed by a structure-based rational optimization, presented two-digit micromolar activity against *M*. *tuberculosis* IspF [73]. Native electrospray ionization-mass spectrometry and docking experiments suggest that bis-sulfonamides such as **39** inhibit IspF by extracting the zinc cation from the active site and by occupying the substrate-binding pocket. This pocket is capable of binding two bis-sulfonamide molecules or their complex with a Zn^2+^ ion [74].

In some bacteria, IspD and IspF activities are performed by two domains of a single IspDF protein. Analytical ultracentrifugation experiments suggested that this bifunctional protein from *Campylobacter jejuni*, as well as IspD and IspF of *E*. *coli*, form complexes with the corresponding IspE proteins [4]. However, electrophoresis on native gel and size exclusion chromatography did not confirm the formation of such assemblies for the enzymes of *E*. *coli* and *B*. *subtilis* [75].

A library of over 100,000 compounds was screened in search of inhibitors of the *Helicobacter pylori* IspD domain of the bifunctional IspDF enzyme. Three inhibitors characterized by submicromolar activity were found (Figure 6). Compound **41** was shown to reduce the growth of *H*. *pylori* to 33% at 19 μM, while **42** and **43** suppressed its growth with MIC of 12.5 μM and 25 μM, respectively. Encouragingly, the latter compound did not reduce the viability of murine fibroblasts in cell culture [76].

Compounds identified as inhibitors of the IspD domain of *H*. *pylori* IspDF were also characterized by a weak inhibitory activity against its IspF domain. Compound **44** reduced the production of MEcPP to 57% at 50 μM as determined by a ^13^C NMR-based assay [76].

### 2.6. Inhibitors of IspG and IspH/LytB

The last two enzymes of the MEP pathway are [4Fe-4S] cluster-dependent reductases. IspG, originally named GcpE [77], is a dimeric enzyme catalyzing the reductive ring opening of MEcPP into HMBPP, the last intermediate of the MEP pathway. IspH, also called LytB, catalyzes the reduction of HMBPP into IPP and DMAPP in an approximate ratio of 6:1 [4].

A difficulty in the design of IspG inhibitors is the drastic conformational change between its substrate-free and substrate-bound forms. In *Thermus thermophilus*, a rotation by about 60° in the angle between the two domains of each monomer of the enzyme is observed [4,78].

**Figure 7:**
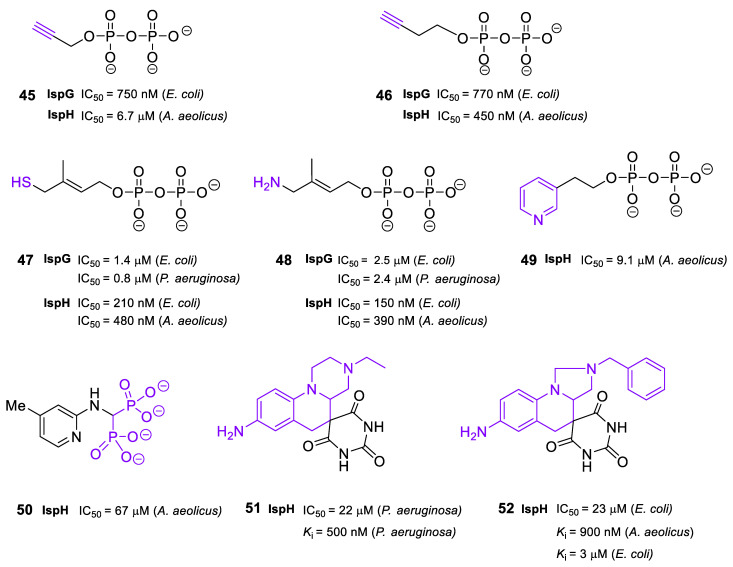
Representative inhibitors of IspG and IspH.

Despite this drawback, several diphosphate compounds containing alkyne, carboxylate, pyridine, and imidazole moieties were tested as inhibitors of IspG. The propargylic and homopropargylic diphosphates **45** and **46** (Figure 7), characterized by the IC_50_ values of 750 nM and 770 nM, respectively, with respect to the enzymes of *E. coli*, were the most potent compounds investigated in this study. The molecules lacking a triple bond were significantly less active, with only three-digit micromolar or millimolar IC_50_ values [79]. Alkynes probably bind an iron atom in the [4Fe-4S] cluster, forming a ferracyclopropene structure [80]. However, this mode of action gives rise to selectivity issues with respect to mammalian enzymes containing iron-sulfur clusters [9]. Nevertheless, the coordination of the cluster in IspG is very specific, with only three iron atoms coordinated by cysteine sulfur atoms and the fourth one binding a glutamate carboxylate. The latter is then replaced by the hydroxyl group of MEcPP [4,77,81].

Besides the druggable pocket identified in the active site of IspH, which contains an unusually high proportion of apolar amino acid, the presence of allosteric sites that could bind larger substrate analog inhibitors was demonstrated [82]. Most of the IspH inhibitors reported in the literature interact with the [4Fe-4S] cluster. Substrate mimics such as the thiol and amino derivatives of HMBPP **47** and **48** (Figure 7) are excellent inhibitors of *E. coli* IspH, with reported IC_50_ values of 210 nM and 150 nM, respectively. The thiol **47** acts as a reversible tight-binding inhibitor, and **48** is a reversible slow tight-binding inhibitor. The inhibitory activity of **48** increases with pH, indicating a more efficient enzyme binding with the non-protonated amino group [83].

Compounds **47** and **48** were also reported to bind the [4Fe-4S] cluster of IspG, exhibiting low micromolar to high nanomolar inhibitory activities against the enzymes of *E. coli* and *P. aeruginosa* [84].

Other IspH inhibitors targeting the [4Fe-4S] cluster with notable activities were diphosphates possessing terminal alkyne and nitrile moieties. The most potent compound investigated was **46** (Figure 7), characterized by a 450 nM IC_50_ value against the IspH of *Aquifex aeolicus*. In the same study, the effects of the substitution of the triple bond with pyridine moieties were investigated. However, these substitutions caused dramatic decreases in inhibitory activity. Compound **49** (Figure 7) was the most active pyridine-based inhibitor tested, with an IC_50_ value of 9.1 μM against the IspH of *A. aeolicus*. Bisphosphate pyridine derivatives were also studied, **50** being the most potent of them with an IC_50_ of 67 μM (*A. aeolicus*) [85].

In silico screening led to the identification of a derivative of a barbituric acid **51** (Figure 7) as a non-diphosphate inhibitor of the *P*. *aeruginosa* IspH with a *K*_i_ value of 500 nM. Its analog **52** is characterized by a *K*_i_ of 900 nM and 3 µM against the enzymes of *A. aeolicus* and *E. coli*, respectively [86].

Targeting IspH alone can lead to the accumulation of HMBPP, which activates γδ T lymphocytes (cf. part II) and thus may overstimulate the immune response [9]. On the other hand, IspH inhibitors targeting its iron–sulfur cluster were reported to suppress the IspG activity as well [9,82,84], and such double targeting may help prevent the emergence of resistance [82].

## 3. MEP Pathway as Immunostimulant Source

### 3.1. Natural Phosphoantigens from the Isoprenoid Biosynthetic Pathways

Phosphoantigens (PAgs) are small non-peptidic phosphorylated molecules capable of activating Vγ9Vδ2 T cells (or Vγ2Vδ2 T cells), a subset of T lymphocytes that express γδ T-cell receptors [87,88]. Displaying innate and adaptative properties, this subset, encountered only in humans, higher primates, and recently found in alpaca cells [89], plays several important roles in the immune system. In particular, it mediates the innate immunity against a wide variety of pathogenic bacteria (e.g., *M. tuberculosis*) and protozoa (e.g., *Plasmodium falciparum*) as well as exerts a broad cytotoxicity against human tumor cells [90,91,92]. Thus, Vγ9Vδ2 T cells are promising targets as cell effectors for cancer immunotherapy.

**Figure 8:**
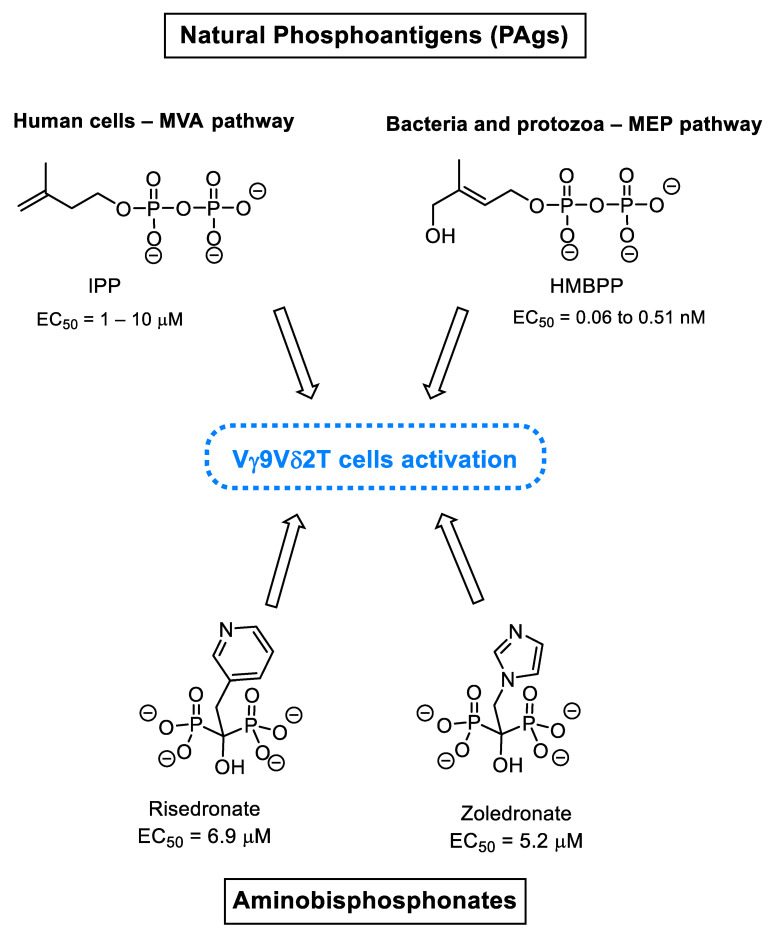
Natural phosphoantigens from the isoprenoid biosynthesis and aminobisphosphonates. MVA: mevalonate; EC_50_: half maximal effective concentration.

The first natural PAg identified was IPP (Figure 8), reported to induce a Vγ9Vδ2 T-cell response in the micromolar range [93]. Its isomer, DMAPP, was also found to exert PAg activity [94], though a recent study concluded that it rather acts as an indirect PAg through a cellular interconversion into IPP catalyzed by isopentenyl diphosphate isomerase (IDI) [95].

The concentrations of IPP in healthy human cells are not sufficient to trigger Vγ9Vδ2 T-cell activation. However, a dysregulation of the mevalonate pathway observed in several tumor cells leads to the accumulation of endogenous IPP and thus induces a Vγ9Vδ2 T-cell response [96]. Strikingly, aminobisphosphonate drugs (e.g., risedronate and zoledronate), originally developed as a treatment for osteoporosis and metastatic bone disease, were found to activate Vγ9Vδ2 T cells using an indirect mechanism. Indeed, they inhibit the farnesyl diphosphate synthase, an enzyme downstream of IPP, and thus increase the intracellular level of IPP and DMAPP among other prenyl metabolites [91,94,96,97,98].

Surprisingly, simultaneously with the discovery of the role of IPP and DMAPP in Vγ9Vδ2 T-cell activation, it was reported that microbes using the MEP pathway to synthesize IPP, such as *M. tuberculosis*, *E. coli,* and *P. falciparum*, produce phosphoantigens that specifically activate Vγ9Vδ2 T cells [10,99]. A natural ligand of microbial origin for the human Vγ9Vδ2 T-cell receptors was then identified as HMBPP [100,101], which turned out to be about 10^4^ times more potent than IPP in activating human Vγ9Vδ2 T cells at picomolar concentrations (HMBPP EC_50_ = 0.39 nM vs. IPP EC_50_ = 10 µM and DMAPP EC_50_ = 20 µM) [98].

Thus, the last intermediate of the MEP pathway plays a dual role, as it is both required for bacterial growth and triggers the host immune response [102].

### 3.2. Synthetic HMBPP Analogs

**Figure 9:**
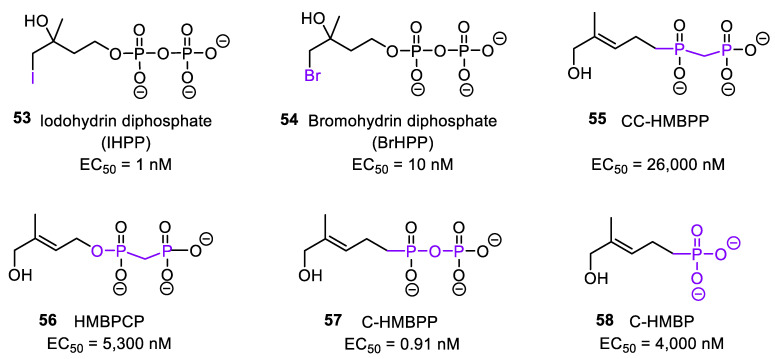
Synthetic phosphoantigens (PAgs).

The difference in activity between HMBPP and DMAPP shows the importance of the hydroxyl group at the C4 position for efficient Vγ9Vδ2 T-cell activation. However, the efficiency of natural HMBPP recognition by Vγ9Vδ2 T cells seems limited as (i) HMBPP is produced in very small amounts in bacteria, especially in slow-growing ones (e.g., *M. tuberculosis*), and (ii) its rapid and strong recognition by Vγ9Vδ2 T cells may seem ineffective for many HMBPP-producing bacteria as they manage to escape the immune response. Therefore, based on the structures of IPP and HMBPP, several PAg agonists for human γδ T lymphocytes have been synthesized (Figure 9) [102,103].

Among these compounds, halohydrin diphosphates mimic the biological properties of natural PAgs and exert an activity similar to HMBPP in inducing Vγ9Vδ2 T-cell proliferation, even though they lack the allylic alcohol moiety. The iodine (IHPP) **53** and bromine (BrHPP) **54** derivatives were the most potent, with EC_50_ values around 1 nM and 10 nM, respectively (Figure 9) [98,103,104]. When combined with low doses of interleukin-2 (IL-2) as co-treatment, BrHPP even demonstrated strong potential in specific activation of Vγ9Vδ2 T cells in clinical trials for the treatment of hematological malignancies and solid tumors [105,106]. However, no clinical developments of BrHPP have been reported in the literature since then [107]. This can be partly due to the rapid degradation of BrHPP in plasma, with a half-life of only a few minutes. Moreover, a decrease in the Vγ9Vδ2 T-cell proliferation in subsequent BrHPP therapy cycles was observed, and increasingly higher doses of BrHPP might thus be required [106,108]. To improve the stability of HMBPP and BrHPP **54** in vivo, derivatives of HMBPP with modifications of the diphosphate moiety have been synthesized.

Replacing the HMBPP diphosphate group, susceptible to chemical or enzymatic hydrolysis, with a diphosphonate group (CC-HMBPP **55**) drastically reduces the capacity of this compound to induce the proliferation of Vγ9Vδ2 T cells. Similarly, a loss of activity is observed when only the second phosphate moiety is substituted by a phosphonate (HMBPCP **56**) (Figure 9). In contrast, the replacement of the first bridging oxygen with a carbon atom (C-HMBPP **57**) results only in a slight decrease in potency compared to HMBPP (EC_50_ of 0.91 nM) while increasing the molecule stability. Using a monophosphate or a monophosphonate moiety (C-HMBP **58**) also abrogated the PAg activity [98,104,109].

Moreover, the impact of the double bond configuration of the isoprene unit has been studied, and the PAgs in their natural *E* configuration turned out to be about 600 times more potent than their *Z*-isomers (*E*-HMBPP EC_50_ = 0.39 nM versus *Z*-HMBPP EC_50_ = 252 nM) [109].

### 3.3. Prodrugs of Phosphoantigens

**Figure 10:**
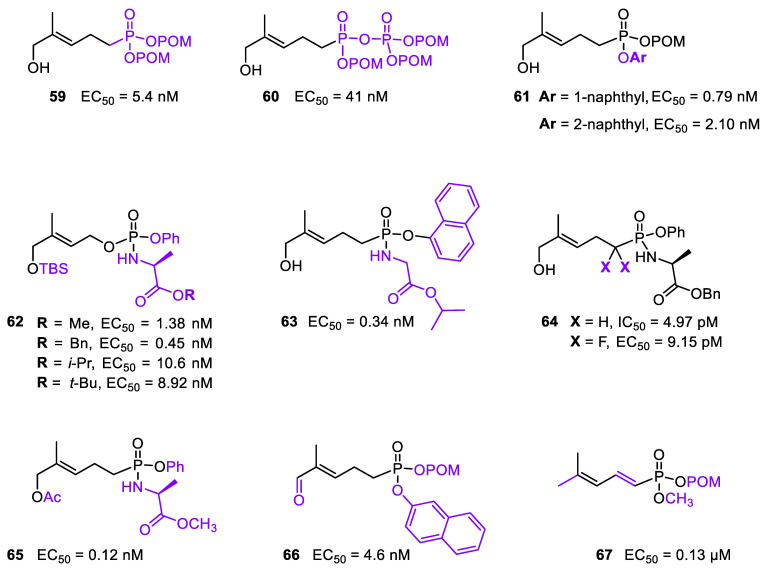
Prodrugs of phosphoantigens.

Except for compound **57** (Figure 9), all attempts to increase the stability of the diphosphate group in HMBPP led to a loss of immunostimulatory activity. This is likely due to the acidic properties and the negative charge of the diphosphate or diphosphonate groups at physiological pH, restraining the movement of HMBPP and its synthetic analogs across the membranes and probably limiting the efficiency of HMBPP-based therapies [98]. To overcome these stability and membrane permeability issues, several PAg prodrugs have been synthesized. The bis- and tris-POM prodrugs of the monophosphonate and diphosphonate derivatives of HMBPP, POM_2_-C-HMBP **59** and POM_3_-CC-HMBPP **60** (Figure 10), displayed potent activation of Vγ9Vδ2 T-cell proliferation in the low nanomolar range (EC_50_ = 5.4 nM and 41 nM respectively). The increased potency of **59** and **60** compared to **58** and **57** suggests that the prodrugs release the free phosphonate forms intracellularly [110,111].

Additionally, the monophosphonate delivered by compound **59** is thought to undergo endogenous phosphorylation to give the potent and stable phosphoantigen C-HMBPP **57**. These results suggest that the PAg charges act as a barrier for both cellular entry and exit and that the binding of PAgs to the butyrophilin transmembrane protein receptor BTN3A1, responsible for their recognition by Vγ9Vδ2 T cells, occurs intracellularly [110,111].

However, the bis-POM prodrugs still display rather short half-lives in human serum as they are fully metabolized after 2 h. Thus, to further increase the prodrug stability, mixed aryl/POM diester prodrugs **61** with 1- and 2-naphthyl moieties have been synthesized. These two prodrugs exhibited high stability in 50% plasma and pico- and nanomolar potencies in activating the Vγ9Vδ2 T-cell proliferation (**61** 1-naphthyl EC_50_ = 0.79 nM; **61** 2-naphthyl EC_50_ = 2.10 nM; HMBPP EC_50_ = 0.51 nM). The prodrug containing the 1-naphthyl moiety was even about 570-fold more potent than HMBPP in stimulating Vγ9Vδ2 T cells for interferon γ production [112,113].

In parallel, the phosphoramidate prodrug approach was applied to the monophosphate derivative HMBP in order to find a monophosphate prodrug as potent as HMBPP. However, due to the poor stability of the P–O bond in the presence of a deprotected hydroxyl group at the C4 position in these prodrugs, only the protected phosphoramidates **62** could be tested. The most potent compound of the series was the benzyl ester derivative, with an EC_50_ value of 0.45 nM [114]. The same strategy was tested on the derivatives of the monophosphonate C-HMBP containing (i) different aryloxy groups, (ii) either glycine or l-alanine ester moieties, and (iii) a CH_2_–P or CF_2_–P bond (compounds **63** and **64**,Figure 10). These prodrugs are highly stable in human plasma and exhibit potent activation of Vγ9Vδ2 T-cell proliferation with EC_50_ values from the mid-picomolar to the femtomolar range, thus displaying an efficiency similar to or even significantly greater than HMBPP [115,116].

Additionally, the protection of the allylic alcohol in aryloxy diester phosphonamidate prodrugs with a biocleavable protecting group such as the acetate ester (compound **65**) increased their potency compared to their free alcohol counterparts and to HMBPP [117].

More recently, modifications at the allylic alcohol of several monophosphonate prodrugs highlighted that the removal of the allylic alcohol abrogates activity; its replacement with an aldoxime simply decreases it, while an aldehyde (compound **66**) is well tolerated as it is reduced intracellularly into the corresponding alcohol. Furthermore, using a diene scaffold instead of the classical isoprene unit, even without the hydroxyl group at C4 as in compound **67**, turned out to be sufficient to induce stimulation of the Vγ9Vδ2 T cells [95,118].

All the prodrugs mentioned above exhibit mild to no toxicity and specifically activate the proliferation of Vγ9Vδ2 T cells. Thus, the phosphonamidate and mixed aryl prodrug strategies seem to provide interesting candidates as new immunotherapeutics for the treatment of cancer and infectious diseases that can be targeted by Vγ9Vδ2 T cells.

## 4. MEP Pathway as a Potential Contributor to Bacterial Cell–Cell Communication

### 4.1. Cell–Cell Communication Signal Molecules in Bacteria

Bacteria are unicellular microbes, but they live communally, inclining us to consider a bacterium more as a component cell of a multicellular organism than a free-living, autonomous biological entity [119]. Thus, bacterial cells have to communicate and cooperate with each other, modifying their behaviors according to changes in their social environment. These interactions are made possible through an extensive repertoire of signaling molecules, also named autoinducers (AIs). These small molecules enable bacterial cells to sense the density of surrounding bacteria and then trigger a coordinated response. Thus, we can consider that bacteria possess a kind of “social intelligence”.

As cell density is a key parameter, these mechanisms are known as quorum-sensing (QS). Interactions between bacteria occur between neighboring cells via soluble molecules or at a distance through volatile apolar organic compounds. These semiochemicals are reported, for example, to inhibit or promote fungal growth [120,121,122]. All these communication mechanisms allow bacteria to detect changes in biotic and abiotic conditions and to respond appropriately to them by switching their phenotypes.

### 4.2. Inter-Kingdom Communication: (S)-4,5-Dihydroxy-2,3-pentanedione and Its Derivatives

These secreted signal small molecules or autoinducers (AIs) demonstrate high chemical structure variability. Besides the acyl homoserine lactones widely described in the literature [123] and often produced by Gram-negative bacteria, numerous other AIs have been identified. One in particular, (*S*)-4,5-dihydroxy-2,3-pentanedione **68** (DPD or AI-2), has been known since 1971 [124] and the discovery of its role in QS in *Vibrio harveyi* by Bassler et al. in 1994 prompted extensive studies of this molecule [125]. DPD is synthesized by the LuxS protein, an enzyme conserved throughout the bacterial kingdom [126]. Hence, LuxS/AI-2 QS system is possibly the first described mechanism of intercellular (intra- and inter-species) communication present in nearly all bacteria. In many of them, it has been linked to pathogenicity [125], and its roles in important physiological functions such as cell signaling, production of virulence factors, stress response, and biofilm formation have been reported [127].

**Scheme 2:**
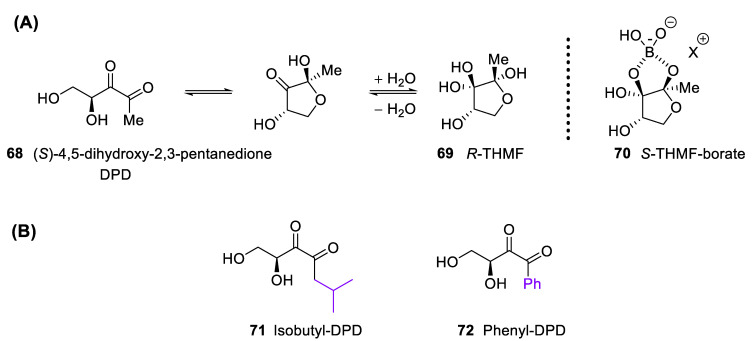
(**A**) DPD as a precursor to AI-2 *S*-(tetrahydroxytetrahydrofuran)-borate diester (*S*-THMF-borate) (**B**) DPD analogs.

The precursor DPD undergoes various rearrangements to form distinct signal molecular structures, which interconvert in a complex equilibrium. Indeed, in an aqueous solution, DPD, a linear five-carbon molecule, is in equilibrium with cyclic stereoisomeric forms (Scheme 2), all these DPD derivatives being referred to as AI-2. Since different chemical species are recognized by different bacteria, studies of AI-2-mediated QS mechanisms have proved challenging. In *V. harveyi*, cyclic DPD derivatives coordinate to boron to form *S*-THMF (tetrahydroxytetrahydrofuran)-borate diester **70,** whereas *Salmonella typhimurium* reacts to *R*-THMF **69** [128]. Unsurprisingly, studies of synthetic DPD analogs have shown how a small structural modification impacted biological activity. These molecules mediate a variety of QS pathways and regulate numerous genes.

The behavior of *P. aeruginosa*, which does not produce DPD, has been shown to be impacted by the addition of DPD, which induced the expression of virulence factor genes [129] and regulated the formation of biofilm and bacterial viability in a dose-dependent manner [130]. In *E. coli*, the DPD analogs, isobutyl-DPD **71** and phenyl-DPD **72** inhibited the maturation of biofilm [131]. In pathogenic *E. coli* O157:H7, proteomic experiments revealed that AI-2 regulated the expression of multiple proteins involved in diverse metabolic processes playing important roles in bacterial virulence, such as adaptation, protection, cell motility, secretion, envelope biogenesis, and protein translation [132].

### 4.3. 4-Deoxy DPD (Laurencione), an MEP Pathway Byproduct

**Scheme 3:**
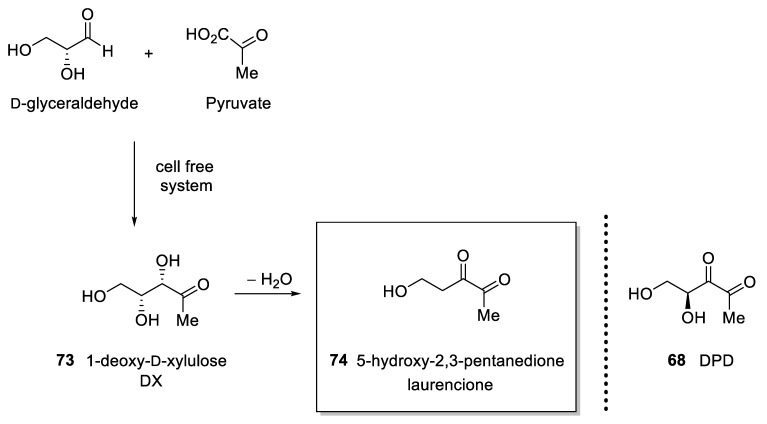
Proposed pathway to laurencione.

The MEP pathway is a major pathway elucidated in the 1990s [133]. During experiments deciphering its seven enzymatic steps [11], when pyruvate and ᴅ-glyceraldehyde were incubated with cell-free systems from *E. coli* or *Klebsiella planticola*, the authors observed not only the production of 1-deoxy-ᴅ-xylulose **73** (DX) but also of a compound identified as laurencione **74**, first isolated in the red alga *Laurencia spectabilis* [134]. Algae are known for producing a huge number of small molecules whose physiological or ecological functions are unknown. Labeling experiments determined the origin of the laurencione carbon atoms indicating that laurencione and DX carbon skeletons resulted from the same condensation reaction and that laurencione was synthesized from DX by elimination of a water molecule (Scheme 3). The dehydratase involved in this reaction is yet unknown.

Strikingly, the molecular structure of laurencione **74** (4-deoxy-DPD) is very similar to that of DPD **68**. Thus, besides the potential role of laurencione in isoprenoid metabolism, one can ask about its functions in cell–cell communication.

In *V. harveyi*, AI-2 signaling controls bioluminescence [126]. In the *V. harveyi* MM30 mutant, unable to synthesize AI-2, the addition of AI-2 restored the quorum signal and luminescence. A similar effect was observed after the addition of laurencione, yet with a 1000-fold lower efficiency [135]. This result showed that the substrate-sensor complex is certainly impacted by the absence of the hydroxyl group at the C4 position.

**Figure 11:**
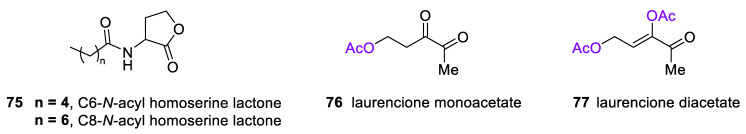
Signal molecules identified in *E*. *coli* JB525 mutant.

In *E. coli* JB525 mutant, which synthesizes an unstable green fluorescent protein in response to C6- and C8-*N*-acyl homoserine lactone **75**, laurencione **74** as well as laurencione mono- and diacetate **76**, **77** (Figure 11) reduced fluorescence by 50% at 600 µM, 150 µM, and 55 µM respectively. Additionally, these three compounds demonstrated anti-inflammatory activities, inhibiting nitric oxide production in lipopolysaccharide (LPS)-stimulated macrophages [136].

From these rather scarce experiments, it is clear that laurencione may be involved in cell communication, probably as an imperfect DPD mimic.

## 5. Conclusions and Remarks

Since the elucidation of the MEP pathway in the early 1990s, its potential as a selective drug target has been the focus of numerous publications. Of the seven enzymes involved, three-i.e., DXS, the best-studied DXR, and IspH-have gained much attention in antimicrobial development. Nevertheless, all the MEP enzymes present drawbacks to the design of inhibitors. As most of the MEP pathway intermediates are phosphorylated, catalytic pockets are polar, and therefore they can accommodate charged phosphate moieties, whereas the ligand design often requires a high degree of lipophilicity. Moreover, targeting general cofactors, such as the IspH Fe–S cluster, is attractive but hazardous, as it may lead to unexpected cytotoxicity. Considering targeting allosteric pockets may seem an elegant way to overcome these downsides, even though it appears to be even more challenging [9]. Additionally, the amino acid sequence of the enzymes from diverse bacteria can differ significantly, making the problem of universal inhibitor design evermore difficult to solve. Moreover, because of the scarcity of crystal structures for IspH and IspG, their inhibitors are tested on enzymes originating from extremophile bacteria, e.g., *T. thermophilus* or *A. aeolicus*. In vivo tests of bioavailable prodrugs of these inhibitors, especially on pathogenic bacteria, would be a welcome addition in the near future.

Another attractive facet of the MEP pathway is the synthesis of a potent phosphoantigen by its antepenultimate enzyme, IspG. While MEP pathway enzymes can be used as targets for antibacterial compounds, this same pathway could trigger γδ T-cell production, which plays critical roles in the early response to invasive pathogensAs bacteria in tumors have been shown to degrade the antitumor agent gemcitabine [137,138], we can capitalize on this dual opportunity to design new prodrugs for use in anticancer treatment which would act on both fronts: neutralizing bacteria as well as inducing an immune response in the host organism.

As all things come in threes, the MEP pathway could still hold surprises. Indeed, the small five-carbon chemical compound identified as a by-product of the pathway deserves consideration. Laurencione, or 4-deoxy-2,3-pentanedione, is structurally similar to the well-known signal molecule DPD, raising questions about its potential roles in quorum-sensing mechanisms or, more broadly, in cell–cell communication. To date, few insights have been made on this matter, but they tend to suggest that laurencione is really involved in these mechanisms. Understanding the mode of action of these signal molecules to disrupt these communications mechanisms may give new opportunities to design innovative antimicrobials.

Besides the synthesis of the small building blocks for isoprenoids, the MEP pathway cellular roles appear to be much more complex than previously thought, especially as the regulation of the MEP pathway is not fully understood. All these observations suggest that “cryptic” functions, i.e., phosphoantigen and laurencione syntheses, work side by side with the main function of the MEP pathway.

With regard to the importance of this pathway, questions worth asking still remain. Could there not be any other by-products of the MEP pathway with important cellular roles? How does regulation of the MEP pathway impact the metabolic flux of all its metabolites?

## Data Availability

Not applicable.
